# Marker-less tracking system for multiple mice using Mask R-CNN

**DOI:** 10.3389/fnbeh.2022.1086242

**Published:** 2023-01-06

**Authors:** Naoaki Sakamoto, Hitoshi Kakeno, Noriko Ozaki, Yusuke Miyazaki, Koji Kobayashi, Takahisa Murata

**Affiliations:** ^1^Animal Radiology, Graduate School of Agricultural and Life Sciences, The University of Tokyo, Tokyo, Japan; ^2^Food and Animal Systemics, Graduate School of Agricultural and Life Sciences, The University of Tokyo, Tokyo, Japan; ^3^Veterinary Pharmacology, Graduate School of Agricultural and Life Sciences, The University of Tokyo, Tokyo, Japan

**Keywords:** mouse behavior, multi-rodent tracking, translational research, psychiatric disorders, Mask R-CNN

## Abstract

Although the appropriate evaluation of mouse behavior is crucial in pharmacological research, most current methods focus on single mouse behavior under light conditions, owing to the limitations of human observation and experimental tools. In this study, we aimed to develop a novel marker-less tracking method for multiple mice with top-view videos using deep-learning-based techniques. The following stepwise method was introduced: (i) detection of mouse contours, (ii) assignment of identifiers (IDs) to each mouse, and (iii) correction of mis-predictions. The behavior of C57BL/6 mice was recorded in an open-field arena, and the mouse contours were manually annotated for hundreds of frame images. Then, we trained the mask regional convolutional neural network (Mask R-CNN) with all annotated images. The mouse contours predicted by the trained model in each frame were assigned to IDs by calculating the similarities of every mouse pair between frames. After assigning IDs, correction steps were applied to remove the predictive errors semi-automatically. The established method could accurately predict two to four mice for first-look videos recorded under light conditions. The method could also be applied to videos recorded under dark conditions, extending our ability to accurately observe and analyze the sociality of nocturnal mice. This technology would enable a new approach to understand mouse sociality and advance the pharmacological research.

## 1. Introduction

Worldwide, approximately 970 million people suffer from psychiatric symptoms, such as anxiety and social difficulties, due to various diseases, including autism spectrum disorders and schizophrenia (GBD 2019 Mental Disorders Collaborators, 2022). In clinical settings, physicians can examine individual cases using verbal information, such as chief complaints and information from family and friends. In contrast, the psychological phenotypes of experimental animals, particularly rodents, are mainly investigated by observing their behavior experimentally, as animals are non-verbal. Therefore, the appropriate evaluation of animal behavior is indispensable for translational research on psychological disorders.

Currently, such studies usually focus on the behavior of a single animal. For example, the anxiety tendency of rodents has been evaluated using thigmotaxis in the open field test and/or elevated plus maze for single mice (Pellow et al., 1985; Simon et al., 1994). However, as humans sometimes feel anxious in social situations, rodent behavior in social groups should be evaluated as well. Another example is the three-chamber test, commonly used to evaluate the sociality of rodents. This test quantifies how long a subject mouse is in contact with stranger and familiar mice (Moy et al., 2004). Nevertheless, only a subject freely explores the three chambers, whereas stranger and familiar mice are trapped in wire cages. This test only evaluates unidirectional communication, which is far from the human clinical situation. Thus, examining multi-rodent behavior can be better for accurate determination of rodent mental status. In addition, since mice are nocturnal, evaluating behavior under dark environments can be useful.

The lack of appropriate tools prevents the evaluation of multi-rodent behavior. Although most researchers have visually evaluated rodent behavior at the present time, tracking multiple rodents with eyes is practically impossible. Hence, tracking tools using specific markers have been developed for multiple animals (Shemesh et al., 2013; Endo et al., 2018; Peleh et al., 2019). For example, Shemesh et al. (2013) stained the mouse body using fluorescent hair dye. Peleh et al. (2019) subcutaneously implanted radio-frequency identification (RFID) chips into mice. Although these methods can accurately track rodents for a long time, we cannot completely exclude the effects of markers on behavior, such as the odor of the staining dye. Maker-less tracking methods are expected to replace the marker-required ones in future.

Recently, deep learning methods have evolved rapidly and been applied to pose estimation and behavior classification tasks in rodents (Mathis et al., 2018; Graving et al., 2019; Pereira et al., 2019; Kobayashi et al., 2021; Ebbesen and Froemke, 2022). Marker-less tracking methods also benefit from the evolution of these technologies (Romero-Ferrero et al., 2019). In 2017, mask regional convolutional neural network (Mask R-CNN) was proposed as a method for identifying the regions of objects in an image (He et al., 2017). This network has been utilized to solve many tasks, such as the detection of lesions in pathological sections (Cao et al., 2019) and organs in medical images (Shieh et al., 2022). Mask R-CNN-based methods can be useful for identifying mouse contour regions.

**FIGURE 1:**
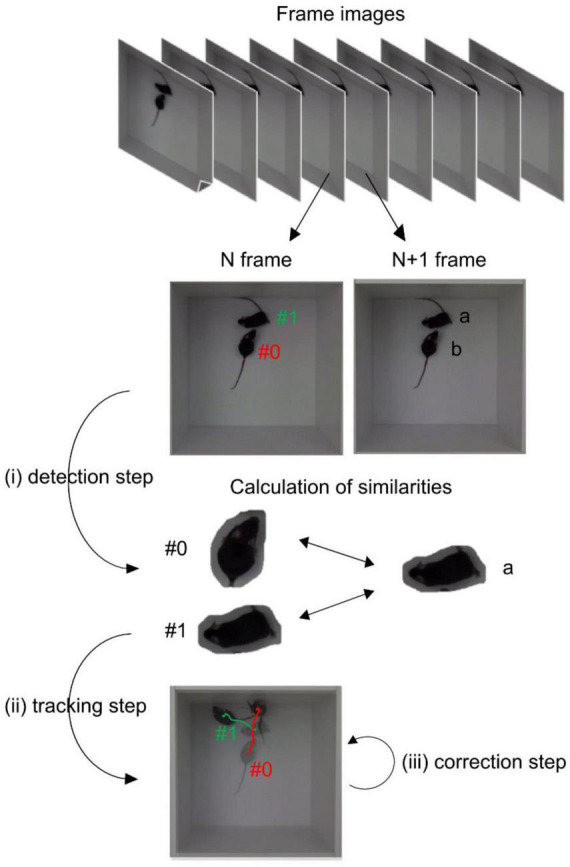
Schematic flow of the proposed method. Mouse contours in each frame were independently identified (detection step). Every mouse was assigned IDs by calculating of similarities (tracking step). Finally, the prediction was semi-automatically corrected (correction step). The background of images for mouse contour regions were removed for visibility.

In this study, we introduced a stepwise method to track multiple mice in top-view videos, without using any markers (Figure 1). First, Mask R-CNN was utilized to identify the mouse contours in each frame image. The acquired contours were then assigned identifiers (IDs) by calculating their similarities between frames using a color-correlogram-based method. Our proposed method successfully tracked two–four C57BL/6 mice in an open-field arena. Additionally, we showed that this method can be applied to videos recorded not only under light conditions but also under dark conditions.

## 2. Materials and methods

### 2.1. Mice

C57BL/6J mice (9–57 weeks old, male and female; Charles River Laboratories Japan, Inc., Yokohama, Japan) were used. All the experiments were approved by the Institutional Animal Care and Use Committee of The University of Tokyo (P19-031). Animal care and treatment were performed in accordance with the guidelines outlined in the Guide to Animal Use and Care of The University of Tokyo.

### 2.2. Video recording

Two, three, or four mice were placed in a white arena (32 cm × 32 cm × 28 cm) and their behavior was recorded for approximately 5 min using a video camera (HDR-CX720V or HXR-NX80, Sony, Tokyo, Japan) set at a height of 110 cm. The recording conditions were as follows: frame rate, 60 Hz; resolution, 1,920 × 1,080 pixels. Mouse behavior under dark conditions were recorded with infrared light and darkroom safe light (the illuminance of room was 2–5 lx). The videos are summarized in Supplementary Table 1. All videos were recorded during daytime (8:00–20:00).

### 2.3. Manual annotation of mouse contours

**FIGURE 2:**
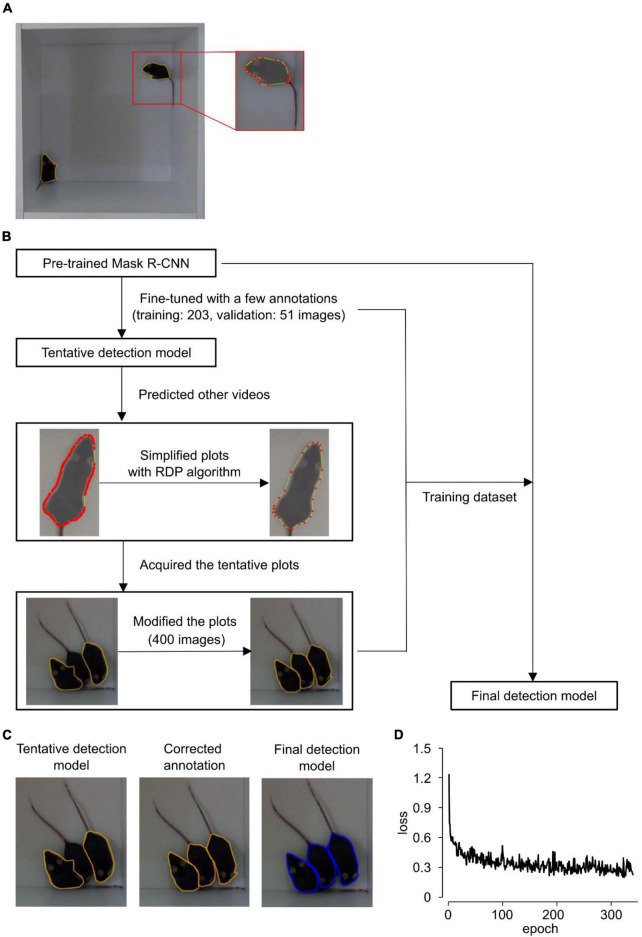
Detection of mouse contours. **(A)** Representative annotated images. Red points and yellow lines indicate vertices and edges of contour. **(B)** Schematic flow of training mask regional convolutional neural network (Mask R-CNN). RDP: Ramer–Douglas–Peucker algorithm. **(C)** Representative images of contour detection. The left, middle and right image show the contours predicted by the tentative detection model, the human-corrected contours, and the contours predicted by the final detection model, respectively. **(D)** The training loss values for the final detection model.

Mouse contours in each frame image were annotated using VGG Image Annotator (Dutta and Zisserman, 2019) (version 2.0.8). Representative examples are shown in Figure 2A. A total of 203 images in video #1 and 51 images in video #2 were manually annotated and used to train and validate the tentative contour detection model. The contours in videos #3 and #4 were predicted using the tentative detection model. Of these, 400 mis-predicted images were selected and corrected manually. These 203 + 51 + 400 images were used training the final detection model. In videos #5, #6, and #7, every 60th frame image was predicted using the tentative detection model. These predicted contours were manually corrected and used as the human annotation for the test dataset.

### 2.4. Contour detection by the tentative model

This study adopted the Mask R-CNN pre-trained with the Microsoft Common Object in Context (COCO) dataset (Lin et al., 2014; He et al., 2017; Waleed Abdulla, 2017) to identify the mouse contours. We fine-tuned Resnet101 stage 4 and the following layers with 203 images and validated it with 51 images for 350 epochs. The hyperparameters were set to default values (Waleed Abdulla, 2017). The model trained for 298 epochs was used as a tentative detection model. The obtained contours were expanded for five pixels and simplified by Ramer–Douglas–Peucker algorithm, where ε = 1.5. The ε value was decided as the number of simplified plots were similar to that of human plots.

### 2.5. Training the final detection model

Four-fold cross-validation was performed to examine the optimal epoch to train the Mask R-CNN (Supplementary Figures 1B, C). The hyperparameters were set to default values (Waleed Abdulla, 2017). We surveyed all validation loss values for 350 epochs and calculated their mean values (Supplementary Figure 1B). Because the lowest mean value was recorded at 341 epochs, we fine-tuned the Mask R-CNN pre-trained with the COCO dataset for 341 epochs with the full training dataset. The trained model is used as the final detection model.

### 2.6. Tracking of identified mice

**FIGURE 3:**
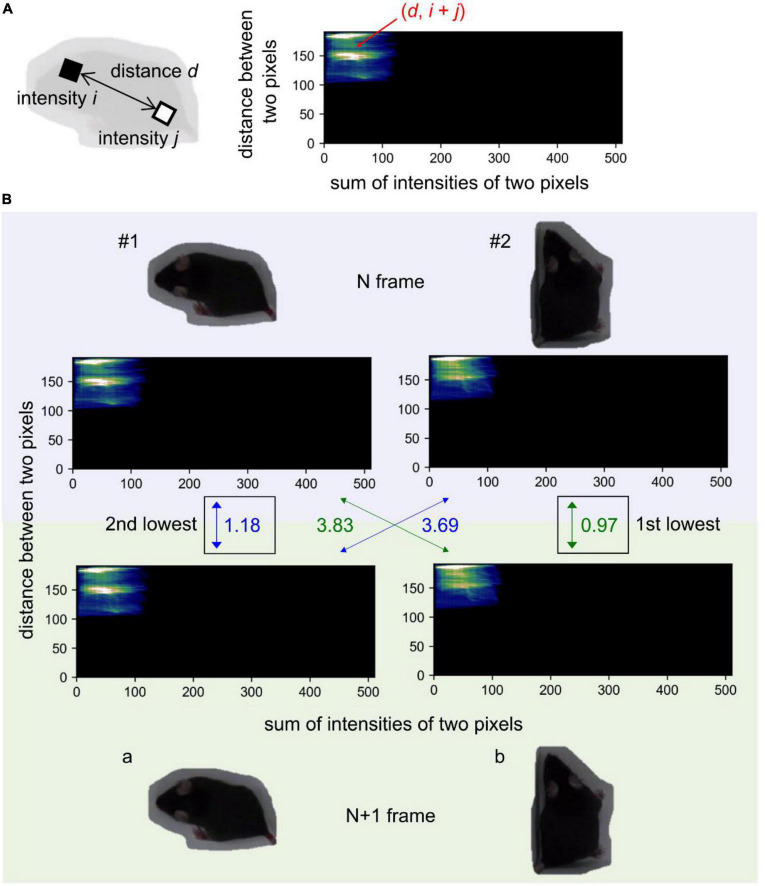
Assigning IDs to identified mice. **(A)** Schematic images of calculating the sum of intensities between two pixels. **(B)** Schematic images of the method to calculate the similarities. The number alongside the arrows indicates the averaged absolute differential values between pair’s histograms at *N* and *N* + 1 frame. Pairs that have first and second lowest values were assigned to same ID. The background of images for mouse contour regions were removed for visibility.

Contour regions were acquired by Mask R-CNN and buffered under the following conditions: dilation (10 iterations, 3 × 3 kernel) and Gaussian blur (7 × 7 kernel). The frame images were cropped along each buffered contour. The buffered images were resized to one-fourth size for reducing computational time and converted to grayscale. Then, the distance and sum of intensities for every pair of pixels in the images were calculated for each contour region (Figure 3A), and two-dimensional histograms were created (Figure 3B). Absolute values of the difference between the pair’s histograms were calculated and averaged to compare the similarities between mouse pairs. The most similar pairs were assigned the same ID. When the number of mice was *n*, *n* contours were assigned IDs and the others were ignored.

### 2.7. Computer hardware and software

The training and prediction of neural networks and other calculations were conducted on a desktop computer equipped with an Intel Core i9-9900KS CPU, 64 GB RAM, and NVIDIA GeForce RTX 2080 Ti. Image processing and training of the neural networks were conducted using the Python.

## 3. Results

### 3.1. Overview of the method

In this study, we recorded top-view videos of multiple mice in an open-field arena. The following steps were used to track multiple mice (Figure 1). First, we divided the videos into frame images and identified the mouse contours in each frame image (detection step). Next, we assigned the ID to each mouse by calculating contour similarities (tracking step). Finally, sporadic misses in mice and unnatural predictions were detected and semi-automatically corrected (correction step).

### 3.2. Contour detection

In the detection step, video files were divided into frames, and individual mouse contour regions in each frame were independently detected using a Mask R-CNN (He et al., 2017; Waleed Abdulla, 2017). Because Mask R-CNN requires a training dataset to identify mouse regions, we had to annotate the contour of each mouse for hundreds of images (Figure 2A). However, such annotation processes are generally labor intensive and time consuming.

In this study, we introduced a stepwise method to reduce the annotation labor (Figure 2B). First, because utilizing models pre-trained with large-scale datasets, known as transfer learning, can efficiently reduce the necessary number of annotations, this study adopted the Mask R-CNN model pre-trained with the Microsoft COCO dataset (Lin et al., 2014) and fine-tuned it with the following simple dataset. The behavior of two C57BL/6 mice in an open-field arena was recorded using a video camera. The video files were divided into frames, and 203 and 51 frames were selected to train the Mask R-CNN and to validate its performance, respectively. During the training, loss values, which show the difference between Mask R-CNN predictions and human annotations, gradually decreased and reached a plateau at approximately 300 epochs (Supplementary Figure 1A). This trained model is hereafter referred to as the “tentative detection model.”

Next, using this tentative detection model, we predicted mouse contours in videos of three–four C57BL/6 mice. We found that some mice were missed and/or mis-plotted when one mouse contacted and/or occluded other mice (Figure 2C). We manually selected 400 mis-plotted frames and modified the contours. Finally, we trained the Mask R-CNN with the 203 + 51 + 400 dataset (details in section “Materials and methods” and Supplementary Figures 1B, C). The training loss values converged successfully (Figure 2D). This model was referred to as “final detection model.” The final detection model successfully predicted difficult images in which a mouse was missed by the tentative detection model (Figure 2C).

### 3.3. Tracking of identified mice

Next, we predicted the mouse contours in each video frame using the final detection model. Because the relationship of detected mice between frames remains unknown, assigning IDs to individual mice is necessary in every frame. First, the mice identified in the first frame were assigned unique IDs. We then calculated the similarities of the detected mice between frames using the color-correlogram-based method, as adopted in idTraker (Pérez-Escudero et al., 2014) (also see section “Materials and methods”), and assigned correct IDs to contours in each frame. Briefly, we calculated the distance and sum of intensities for every pair of pixels in the images of each counter region (Figure 3A) and created two-dimensional histograms (Figure 3B). Then, to compare the similarities between mouse pairs, absolute differential values between pair histograms were calculated and averaged. The values for all pairs were sorted in ascending order, and the corresponding IDs were assigned in order (Figure 3B).

### 3.4. Corrections of predictive errors

We created videos that displayed the geometric centers of contours and assigned IDs for each mouse, and checked their predictive performance. There were two types of problematic predictions: (i) sporadic misses (Supplementary Video 1; mouse ID #3 at 0:00:02) and (ii) irreversible ID switches (Supplementary Video 2; mouse ID #0 and #1 at 0:00:00). Sporadic misses were defined as cases in which the final detection model underestimated the number of mice per frame. Irreversible ID switches were defined as cases where the assigned IDs were accidentally but persistently interchanged between individual mice. The proportion of sporadic misses in all frames tended to increase according to the number of mice in the videos, and an irreversible ID switch was found in one video (Table 1).

**TABLE 1:** Corrected errors and warnings in the training dataset.

Video no.	Number of mice	Misses/All frames (%)	Tracking warning (count)	Irreversible ID switches (count)
1	2	0.46	0	0
2	2	0.21	0	0
3	3	1.00	1	1
4	4	1.85	4	0

To address these problems, semi-automated processing was applied to predictions. First, sporadic misses were automatically fulfilled with previously predicted IDs (Supplementary Figure 2A, Video 3). Next, to identify the ID switches, the distances between the geometric centers of all pairs of continuous frames were calculated (Supplementary Figure 2B). When the distances between the coordinates of mouse ID *i* at frame *N* and *N* + 1 were not the shortest among those of all ID pairs, we set our method to offer “tracking warning.” Consequently, five tracking warnings were identified in the predicted videos (Table 1). One of the tracking warnings successfully identified irreversible ID switching, whereas the other three warnings were irrelevant to irreversible ID switching. Here, we manually exchanged IDs when switching occurred (Supplementary Video 4). These semi-automated correction steps were applied to the following predictions.

### 3.5. Evaluation of the performance

A test dataset was created to evaluate the performance of the proposed method. The contours of the videos of two, three, and four C57BL/6 mice were acquired using the tentative detection model. These contours were checked and corrected by humans every 60th frame and used as manually annotated contours.

**FIGURE 4:**
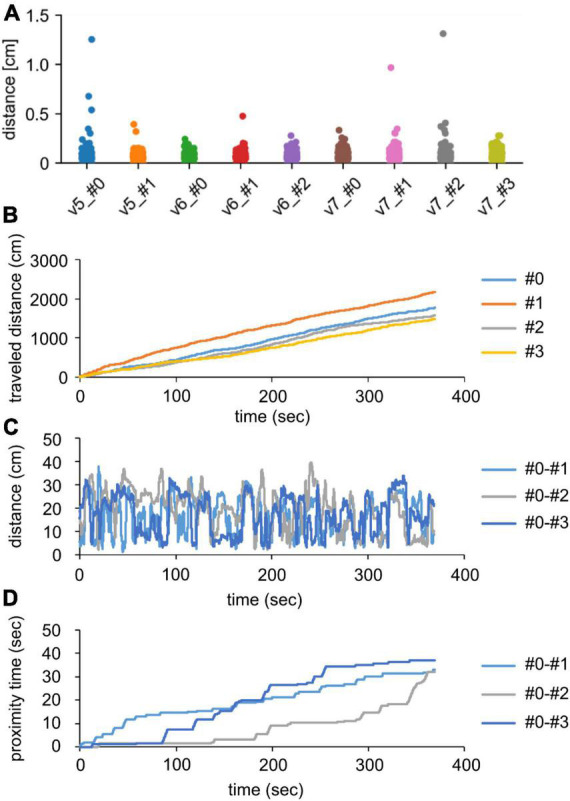
Evaluation of the proposed method. **(A)** Distance between geometric centers of mouse contours annotated by humans and those predicted by the proposed method. v5, v6, and v7 indicate the video no. 5, 6, and 7, respectively (see Supplementary Table 1). These videos were recorded under light conditions. #0, #1, #2, and #3 indicate the individual mouse IDs. **(B)** Cumulative traveled distances of individual mice in the video no. 7. **(C)** Distances between #0 mouse and other mice in the video no. 7. **(D)** Cumulative proximity time between #0 mouse and other mice in video no 7. We defined “proximate” when distance between geometric centers of each pair was less than 6 cm.

Next, each video was predicted using the established method. In the correction step, 0.03–0.82% of the frames per video were compensated as sporadic misses. Additionally, three tracking warnings were proposed, although irreversible ID switches were not observed (Table 2). After the corrections, we evaluated the predictive performance by calculating the distances between the geometric centers of the predicted and manually annotated contours every 60th frame. All values were less than 1.5 cm, and 99.8% values were less than 0.5 cm (Figure 4A). As the body size of the recorded mice was approximately 8 cm (Supplementary Figure 3), these results suggest that our method precisely predicts the coordinates of geometric centers.

**TABLE 2:** Corrected errors and warnings in the test dataset and night application.

	Video no.	Number of mice	Misses/All frames (%)	Tracking warning (count)	Irreversible ID switches (count)
Day	5	2	0.03	0	0
	6	3	0.16	0	0
	7	4	0.82	3	0
Night	8	2	0.79	6	0
	9	3	0.78	7	1
	10	4	4.37	26	1

In addition, we examined whether our system could be applied to videos recorded under dark conditions that were not used for the training dataset. Videos of two, three, and four C57BL/6 mice in an open field arena were recorded with infrared light and predicted using the established method. In the correcting step, 0.79–4.37% of the frames per video were compensated as sporadic misses. and 42 tracking warnings were proposed, which identified irreversible ID switches twice (Table 2). These results were comparable to those of the videos of two and three mice recorded under light conditions. However, the predictive performance for the video of the four mice under dark conditions was slightly inferior to that under light conditions. Adding a training dataset of images recorded under dark conditions can improve performance. Representative performances under light and dark conditions after the corrections are shown in Supplementary Videos 5, 6.

### 3.6. Analysis of mouse activities

Finally, we showed the typical methods to evaluate activities and social interactions using the predicted coordinates of the video recorded under light conditions. Our method enabled us to calculate the cumulative travel distances of the individual mice (Figure 4B). This might reflect the characters in social groups such as “quiet” and “restless.” In addition, sociality can be evaluated by calculating the distance between individuals and the duration during which the mice are located close together (Figures 4C, D). These analyses can reveal whether a mouse accompanies other mice. In conclusion, our established method can help us analyze the behavior of social groups.

## 4. Discussion

Evaluating the social behavior of rodents is indispensable for research on psychiatric disorders. Automated tracking methods are required because we cannot simultaneously follow multiple rodents with eyes. In this study, we established a marker-less tracking system for multiple mice using Mask R-CNN and a color-correlogram-based method.

The current methods for evaluating social behavior among rodents have limitations. Rodents are usually bred in groups and exhibit social interactions with other individuals under both light and dark conditions. These interactions in a social group can reflect the sociality of rodents and are expected to be useful indicators in translational research. However, the limitations of human observations and technological tools have prevented the evaluation of natural interactions among multiple rodents. Researchers usually assess unidirectional social behavior with a three-chamber test (Moy et al., 2004) and/or interactions between only two individuals using a social interaction test (File and Hyde, 1978) under light conditions. Our proposed method can simultaneously track two–four C57BL/6 mice in an open field arena under both light and dark conditions. As shown in Figures 4B–D, the predicted tracking data enabled us to analyze activities in social groups and social proximity. Endo et al. (2018) revealed that social proximity was influenced by breeding conditions during the development phase of mice, which indicates that the analysis of social proximity is important for assessing sociality. The established method can be used to discover novel findings that conventional methods could not.

This study applied the step that semi-automatically correct predictive errors: sporadic misses of mice and irreversible ID switches. Given that these errors significantly affect the analyses of mouse behavior, this correction step is important to understand mouse interactions precisely. In contrast, since it is also true that we cannot check numerous errors one by one, the pre-corrected predictions should have low errors. As shown in Table 2, the errors under dark conditions tend to be more than those under light conditions, especially as the number of mice increased. Since the training dataset of Mask R-CNN did not contain any images recorded under dark conditions, adding their images to the training dataset can contribute to the improvement of performances, and further reduce the burden to check errors in the correction step.

The established method enabled us to conduct different types of experiments. The simplest application is to screen social-deficient symptoms in psychiatric model mice, such as autism spectrum disorders and depression (Kazdoba et al., 2016; Wang et al., 2017). The new screening system would more precisely assess the therapeutic effects of drug candidates than the classical methods. In addition, since psychiatric disorders sometimes affect not only patients but also people living together (Benazon and Coyne, 2000), another application is to investigate how the psychiatric phenotypes of psychiatric model mice affect those of co-housed healthy mice. Similar to humans, rodent behavior is influenced by the emotions of others (Keysers et al., 2022). Boyko et al. (2015) reported that healthy rats co-housed with depressed rats for 5 weeks exhibited depressive-like behavior. In contrast, Wu et al. (2021) showed that mice groomed stressed cagemates more than control ones and relieved stressed ones. We expect that this kind of experiment will contribute to the consideration of appropriate interactions between patients and those living together.

Over the last few decades, tracking methods for multiple animals have been developed (Shemesh et al., 2013; Pérez-Escudero et al., 2014; Endo et al., 2018; Peleh et al., 2019; Romero-Ferrero et al., 2019; Panadeiro et al., 2021). As adopted in this study, tracking methods often consist of two steps: detection and tracking. The most classical method for detecting an animal’s region is the thresholding of frame images (Panadeiro et al., 2021). Although this method has the advantage of high computational speed, it is vulnerable to changes in recording conditions, such as light. Thus, deep-learning technologies that can robustly identify object positions and/or contours have replaced the classical method. Barreiros et al. (2021) utilized You only look once version 2 (YOLOv2), a neural network for object detection, to track multiple zebrafish. In this study, we also showed that Mask R-CNN successfully identified the mouse contour regions. Our results were consistent with those of Le et al. (2021). More recently, pose estimation toolkits for multiple animals using deep learning have been proposed, such as multi-animal DeepLabCut (maDLC) and social LEAP (SLEAP) (Lauer et al., 2022; Pereira et al., 2022). These innovative methods can be used to analyze social behavior in detail. Whether our methods can expand functions, such as pose estimation, will be investigated in future work.

In conclusion, we established a marker-less tracking system for multiple mice and showed that this system can be used under both dark and light conditions. The development of these techniques will allow researchers to assess animal sociality in a natural environment and observe the phenotypes of animals that have been previously missed. The proposed method would be helpful to understand similarities and differences between mouse and human sociality, and advance translational research on psychiatric disorders.

## Data availability statement

The original contributions presented in this study are included in this article/Supplementary material, further inquiries can be directed to the corresponding author.

## Ethics statement

The animal study was reviewed and approved by the Institutional Animal Care and Use Committee of The University of Tokyo (P19-031). Animal care and treatment were performed in accordance with the guidelines outlined in the Guide to Animal Use and Care of The University of Tokyo.

## Author contributions

TM, NS, and HK designed and managed the project. NS and HK performed the experiments. NS, NO, YM, and KK analyzed the data. NS, KK, and TM drafted the manuscript. All authors have read and approved the final manuscript and have agreed to be accountable for all aspects of the work.
