# Supplementary material for: Marker-less tracking system for multiple mice using Mask R-CNN
Source: Front Behav Neurosci. 2023 Jan 6;16:1086242. doi: 10.3389/fnbeh.2022.1086242 (PMC9853548; doi:10.3389/fnbeh.2022.1086242)
Supplement: Supplementary file 1 [file Data_Sheet_1.docx]

**Supplementary Table 1 Video list**

|  | Video No. | Number of mice | light /dark | annotated images for training | |
| --- | --- | --- | --- | --- | --- |
|  |  |  |  | tentative detection model | final detection model |
| training dataset | 1 | 2 | light | 203 images for training | 203 images for training |
|  | 2 | 2 | light | 51 images for validation | 51 images for training |
|  | 3 | 3 | light | - | 200 images for training |
|  | 4 | 4 | light | - | 200 images for training |
| test dataset | 5 | 2 | light | - | - |
|  | 6 | 3 | light | - | - |
|  | 7 | 4 | light | - | - |
| application | 8 | 2 | dark | - | - |
|  | 9 | 3 | dark | - | - |
|  | 10 | 4 | dark | - | - |

**Supplementary Fig. 1**


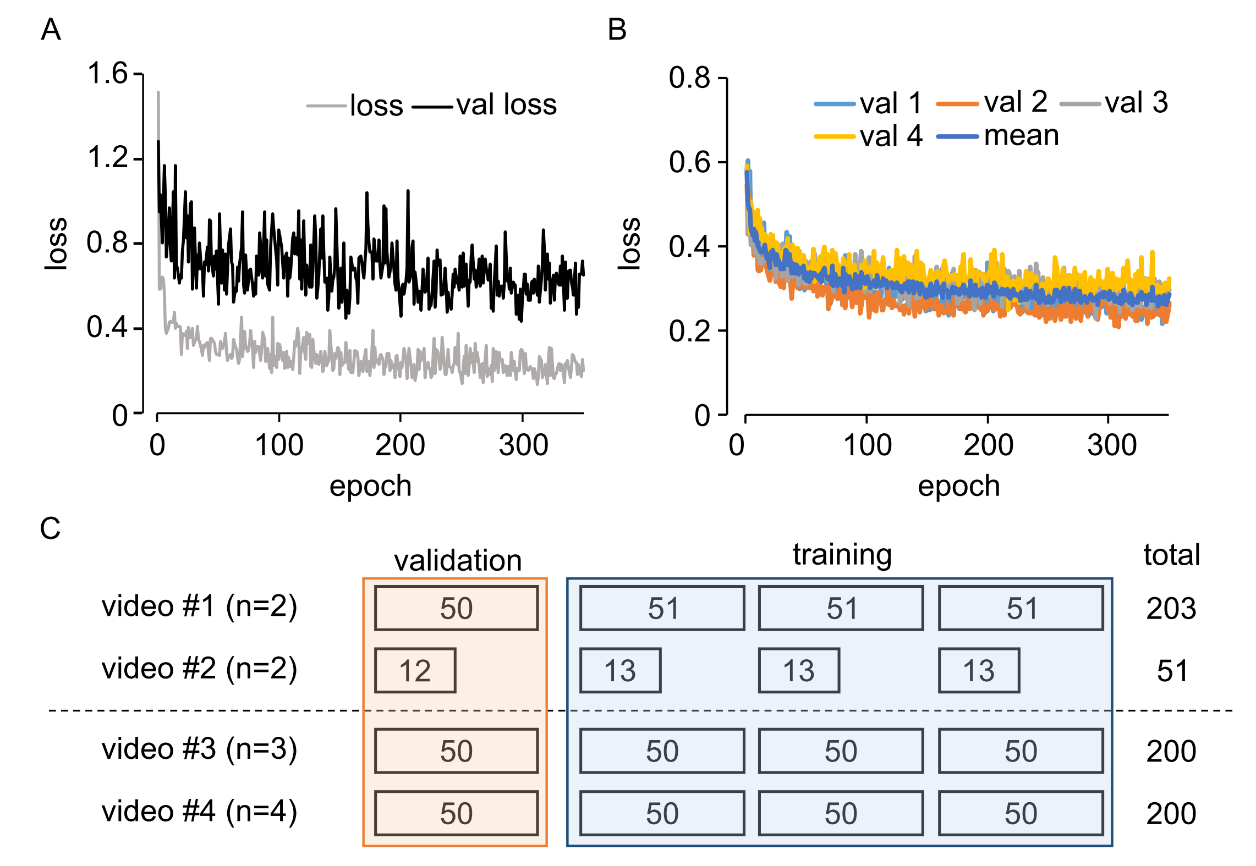


**Supplementary Fig. 2**


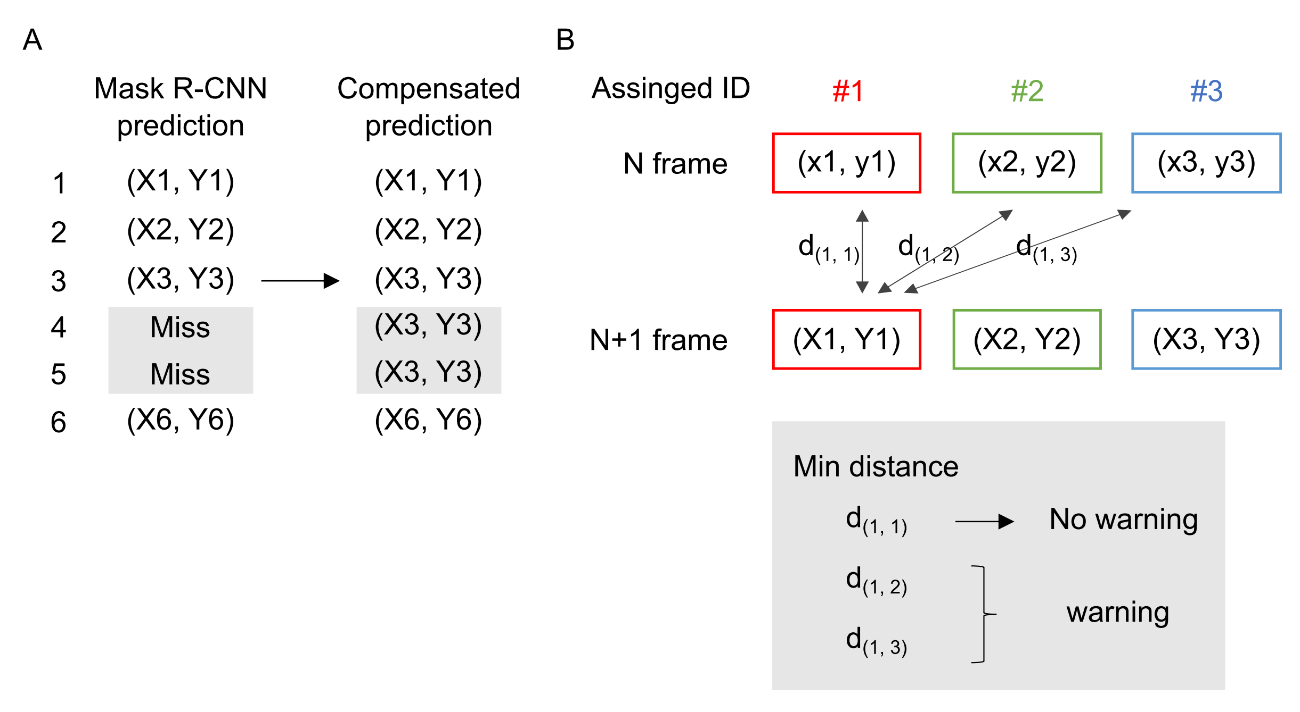


**Supplementary Fig. 3**


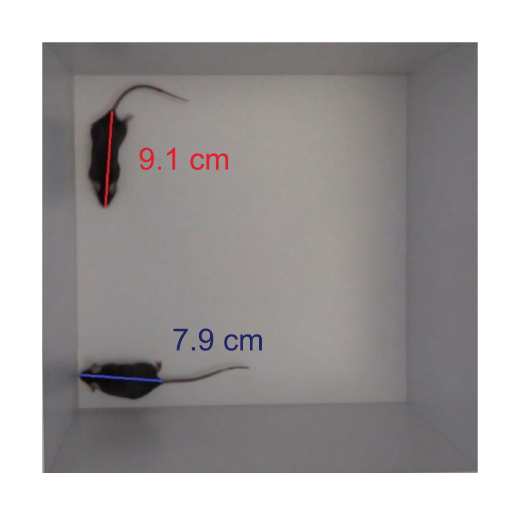


**Figure legends**

**Supplementary Fig. 1 Training Mask R-CNN**

(A) Training and validation losses during training Mask R-CNN for the tentative detection model (B) Validation losses during four-fold cross validation. (C) Representative combination of training and validation dataset in four-fold cross validation. Each video was divided to four sub datasets. Three of them were used as training and the other was used as validation.

**Supplementary Fig. 2 Correction of the predictions**

(A) Schematic images of compensating sporadic misses. (B) Schematic images of the method to output the tracking warning. d_(i,j)_ indicates the distance between the geometric center of ID *i* at *N* frame and *j* at *N+1* frame.

**Supplementary Fig. 3 Representative mouse body size**

Each colored line and value indicate distance from nose to tail.

**Supplementary Video. 1-1 Sporadic misses**

**Supplementary Video. 1-2 Irreversible ID switches**

**Supplementary Video. 2-1 Compensated sporadic misses**

**Supplementary Video. 2-2 Corrected irreversible ID switches**

**Supplementary Video. 3 Representative predictive performance for light video**

**Supplementary Video. 4 Representative predictive performance for dark video**

All supplementary videos were cropped along the arena and trimmed for corresponding events.
